# High-pressure X-ray photon correlation spectroscopy at fourth-generation synchrotron sources

**DOI:** 10.1107/S1600577524001784

**Published:** 2024-04-10

**Authors:** Antoine Cornet, Alberto Ronca, Jie Shen, Federico Zontone, Yuriy Chushkin, Marco Cammarata, Gaston Garbarino, Michael Sprung, Fabian Westermeier, Thierry Deschamps, Beatrice Ruta

**Affiliations:** a Institut Néel, Université Grenoble Alpes and Centre National de la Recherche Scientifique, 25 rue des Martyrs – BP 166, 38042 Grenoble, France; b European Synchrotron Radiation Facility, 71 avenue des Martyrs, CS 40220, 38043 Grenoble, France; c DESY, Notkestraße 85, D-22607 Hamburg, Germany; d University of Lyon, Université Claude Bernard Lyon 1, CNRS, Institut Lumière Matière, F-6922 Villeurbanne, France; University of Malaga, Spain

**Keywords:** X-ray photon correlation spectroscopy, fourth-generation synchrotron sources, high-pressure XPCS, complex systems, metallic glasses, supercooled liquid states, high-pressure sample environments

## Abstract

A new experimental setup combining X-ray photon correlation spectroscopy (XPCS) in the hard X-ray regime and a high-pressure sample environment has been developed to monitor the pressure dependence of the internal motion of complex systems down to the atomic scale in the multi-gigapascal range, from room temperature to 600 K. The high flux of coherent high-energy X-rays at fourth-generation synchrotron sources solves the problems caused by the absorption of diamond anvil cells used to generate high pressure, enabling the measurement of the intermediate scattering function over six orders of magnitude in time, from 10^−3^ s to 10^3^ s. The constraints posed by the high-pressure generation such as the preservation of X-ray coherence, as well as the sample, pressure and temperature stability, are discussed, and the feasibility of high-pressure XPCS is demonstrated through results obtained on metallic glasses.

## Abstract 2

The development of an experimental setup to probe the dynamics of complex systems under high pressure in the gigapascal regime is presented. Feasibility is demonstrated on metallic systems both in glassy and in supercooled liquid states.

## 1. Introduction

Amorphous materials are ubiquitous in our daily life. Although they lack a well defined long-range microscopic structure, many hard and soft glassy systems feature common microscopic relaxation processes which control the evolution of their macroscopic properties (Ngai, 2011 ▸). Examples are proteins in crowded media (Bin *et al.*, 2023 ▸; Begam *et al.*, 2020 ▸; Roosen-Runge *et al.*, 2011 ▸; Foffi *et al.*, 2014 ▸), polymers (Conrad *et al.*, 2015 ▸; Arbe *et al.*, 1998 ▸; Cangialosi, 2014 ▸), clays (Angelini *et al.*, 2014 ▸; Shalkevich *et al.*, 2007 ▸; Jabbari-Farouji *et al.*, 2008 ▸; Nigro *et al.*, 2020 ▸), viscous alloys (Busch *et al.*, 2007 ▸; Wang, 2019 ▸; Gallino, 2017 ▸), network glasses (Sidebottom, 2015 ▸; Micoulaut, 2016 ▸) and pharmaceutical compounds (Wang *et al.*, 2021 ▸; Rodríguez-Tinoco *et al.*, 2016 ▸). Among the large family of disordered systems, structural glasses play a key role, often being considered as archetypes of materials far from thermodynamic equilibrium. A comprehensive microscopic theory of their amorphous state, a long-sought quest in material physics (Gibbs & DiMarzio, 1958 ▸; Adam & Gibbs, 1965 ▸; Barrat & Hansen, 2003 ▸), depends on an accurate description of the system dynamics, *i.e.* its internal motion, from the inter-constituent length scale up to the macroscopic regime, and over the complete timescale of the ongoing relaxation processes, *i.e.* from picoseconds to seconds (Egami & Ryu, 2020 ▸).

This description of the state of the system needs to go through the determination of high-order correlation functions that go beyond the spatially averaged structure factor or pair distribution function. Experimentally, higher-order correlation functions and thus dynamical properties of complex systems at nanometric and atomic length scales can be obtained by means of the X-ray photon correlation spectroscopy (XPCS) technique (Sutton, 2008 ▸; Madsen *et al.*, 2016 ▸; Shpyrko, 2014 ▸; Lehmkühler *et al.*, 2021 ▸). XPCS quantifies the temporal intensity correlation of fluctuating speckles generated by the scattering from a disordered system, *g*_2_(*q*,Δ*t*), to obtain information on the internal dynamics of the system by the Siegert relation,

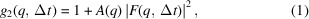

where 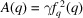 depends on the non-ergodicity parameter *f*_*q*_(*q*) and the degree of coherence γ (contrast) of the experimental geometry. *F*(*q*,Δ*t*) is the intermediate scattering function (ISF), *i.e.* the Fourier transform of the Van Hove correlation function (Madsen *et al.*, 2016 ▸; Shpyrko, 2014 ▸; Lehmkühler *et al.*, 2021 ▸).

To date, experimental studies of the relaxation dynamics in glass-formers have focused on the temperature dependence of the atomic motion (Amini *et al.*, 2021 ▸; Wang *et al.*, 2015 ▸), its response to external mechanical and thermal stresses (Zhou *et al.*, 2020 ▸; Küchemann *et al.*, 2018 ▸; Luo *et al.*, 2020 ▸; Das *et al.*, 2020 ▸), or to its interaction with intense X-ray beams in the case of oxide and chalcogenide glasses (Martinelli *et al.*, 2020 ▸; Alfinelli *et al.*, 2023 ▸; Dallari *et al.*, 2023 ▸; Chushkin, 2020 ▸; Li *et al.*, 2022 ▸; Pintori *et al.*, 2019 ▸).

Among the different properties of interest affecting the dynamics of glasses and liquids, density plays a major role. The viscosity of glass formers strongly depends on the density in molecular liquids and polymers (Grocholski & Jeanloz, 2005 ▸; Kondrin *et al.*, 2012 ▸; Paluch *et al.*, 2007 ▸), which in turn affects the glass transition (Paluch *et al.*, 2001 ▸; Niss & Alba-Simionesco, 2006 ▸; Niss *et al.*, 2007 ▸). Density also affects the relaxation phenomena deep in the glassy state: physical ageing, that is the slow relaxation of the glass towards a metastable equilibrium state, was shown to be mediated by density-driven rearrangements releasing residual stresses and medium-range ordering processes not affecting the local density in metallic glasses (Giordano & Ruta, 2016 ▸).

Transitions between different amorphous states have also been reported in many out-of-equilibrium materials, where pressure can drive the system from a low- to a high-density amorphous state with different physical properties (Tanaka, 2020 ▸; Machon *et al.*, 2014 ▸; Zhang *et al.*, 2010 ▸). These liquid–liquid or glass–glass polyamorphic transitions appear in all kinds of systems, including the canonical case of water (Mishima, 2021 ▸; Amann-Winkel *et al.*, 2013 ▸), covalent (Machon *et al.*, 2014 ▸), ionic (Wojnarowska *et al.*, 2022 ▸) and metallic systems (Sheng *et al.*, 2007 ▸). Though structural studies under pressure have been reported, to date, very little is known on the evolution of the relaxation dynamics during pressure-induced polyamorphic transitions due to the experimental challenge behind the use of high pressure (HP) sample environments for dynamical studies, and previous work has focused mainly on temperature-induced liquid–liquid transitions (Perakis *et al.*, 2017 ▸; Amann-Winkel *et al.*, 2013 ▸; Hechler *et al.*, 2018 ▸).

In the case of XPCS, the relatively low coherent flux at photon energies (*E*) higher than *E* > 15 keV in the majority of third-generation synchrotrons limited the use of bulky sample environments such as those necessary for studies under HP. Owing to the advent of fourth-generation synchrotrons, such as the extremely brilliant source ESRF (ESRF-EBS), it is possible to deliver high-coherence X-ray fluxes also at high photon energies (*E* ≃ 20 keV), solving the absorption issue related to bulky sample environments and thereby opening the possibility of time-resolved, high-quality HP-XPCS in glassy systems, unlocking new fields of investigation (Cornet *et al.*, 2023 ▸; Zhang *et al.*, 2023 ▸). Based on the first HP-XPCS measurements, this paper addresses the different requirements and main issues encountered at the experimental level to obtain reliable dynamical data under HP and high temperature (HT) with atomic-scale XPCS.

## 2. Experimental

### 2.1. Signal-to-noise ratio, absorption and coherence at fourth-generation synchrotron sources

The signal-to-noise ratio (SNR) in an XPCS experiment scales linearly with the average intensity 〈*I*(*q*)〉 and the square root of the minimum sampling time τ_min_ and measurement time *t*, and is defined as

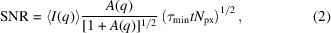

where *N*_px_ is the number of pixels of the detector used to average the correlation function (Jankowski *et al.*, 2023 ▸). Equation (2) has three remarkable consequences. SNR has a linear dependence on the coherent flux (and not with its square root, as for standard intensity measurements) and any gain factor *n* will readily translate into a possible minimum sampling time τ_min_ smaller by a factor of *n* ^2^ for the same SNR. On the other hand, decreasing the intensity by a factor α translates into an increase of the total acquisition time *t* by α^2^ for a similar SNR. An additional flux reduction by an absorbing sample environment implies even longer acquisition times, rapidly exceeding laboratory timescales.

For synchrotron radiation (SR) sources, the coherent flux *F*_c_ relates to the source brilliance *B*, as *F*_c_ = *B*(λ/2)^2^, where *B* quantifies the emission of photons per unit time, unit area, unit solid angle and band-width. The λ^2^ dependence causes a strong decrease in coherent flux at high energies, which can be compensated by a large brilliance. Practically, this means that, at third-generation SR sources, XPCS was usually applied with a coherent flux on the order of 5 × 10^9^ photons s^−1^ to 5 × 10^10^ photons s^−1^ in the 8–11 keV energy range [beamlines ID10 at ESRF (Favre-Nicolin *et al.*, 2017 ▸), 8-ID-E at APS (Jiang *et al.*, 2023 ▸) and P10 at PETRA III (Sprung *et al.*, 2023 ▸)]. This is an energy domain where absorption of even a few millimetres of structural material is prohibitive to obtain a satisfying SNR over a reasonable timescale. Coherent X-rays are still available at higher energies (Frost *et al.*, 2023 ▸), albeit at such substantially reduced fluxes that, in some cases, the use of the bulky sample environment used for HP generation becomes non-feasible.

The advent of the fourth-generation SR sources like the ESRF-EBS led to a considerable jump in brilliance and therefore in coherent flux (Favre-Nicolin *et al.*, 2017 ▸) by up to two orders of magnitude owing to the reduction of the horizontal emittance of the electron beam (Raimondi *et al.*, 2023 ▸). As an example, Table 1 ▸ lists the brilliance from the X-ray beam produced by the U27 undulator at the ID10 beamline at EBS-ESRF, computed from the experimentally measured spectral flux [with primary slits (at 27.2 m from the source) open to 0.15 mm × 0.15 mm] (Zontone *et al.*, 2010 ▸) over the first and third harmonics. Pre-EBS, high-β electron source reference values are reported for comparison. A 70× gain is observed at the third harmonic at 21 keV (Jankowski *et al.*, 2023 ▸), resulting in an available coherent flux of about 10^11^ photons s^−1^, exceeding even the maximum coherent flux previously available from the full ID10 high-β straight section (consisting of two U27 undulators and one U35 undulator) at 8 keV (Jankowski *et al.*, 2023 ▸).

In Table 1 ▸, we also report the transmission through a typical diamond anvil cell (DAC), the apparatus often used to generate HP. This transmission, calculated through two 1.7 mm-thick diamonds, increases from 0.5% to 68% from 8 keV to 21 keV, and shows how the energy shift opens the door to HP measurements based on coherent scattering.

In addition to the increased incident coherent flux, several other constraints exist to perform HP-XPCS, including the preservation of the coherence through the sample environment, the pressure and temperature stability, the absence of shear stress on the sample at HP and the impact of the intense irradiation on the sample environment. These will be discussed later.

### 2.2. Setup and feasibility

**Figure 1:**
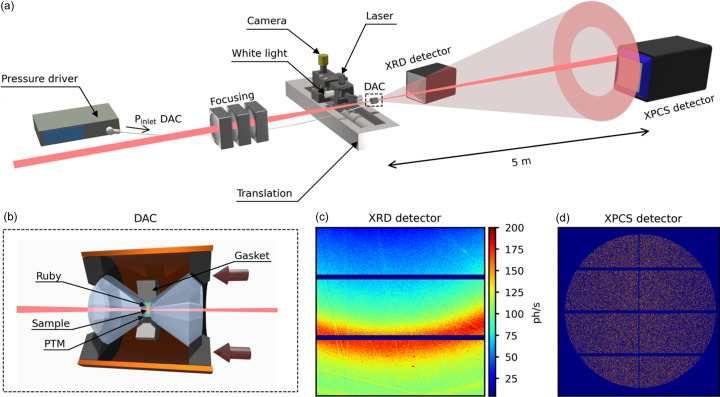
Schematic of the wide-angle HPHT-XPCS experimental setup (*a*), with a sketch of the DAC (*b*), and raw images from a PILATUS 300 K detector dedicated to diffraction (*c*) and an EIGER 4M to XPCS (*d*). The integration time for the diffraction and XPCS images are 60 s and 0.1 s, respectively. A simplified colour code is used for the XPCS detector, where all active (non-zero) pixels are shown, corresponding to 1 (96.67%), 2 (3.25%) or 3 (0.08%) impinging photons. All the elements necessary for HP(HT)-XPCS are labelled.

The experimental setup used to probe the effects of pressure on the internal atomic dynamics of disordered systems is schematized in Fig. 1 ▸(*a*). The incoming beam is produced by three undulators, followed by a first collimation with high-power slits and a Pd-coated double mirror at grazing incidence to supress higher harmonics. The X-ray beam is made monochromatic by a cryo-cooled channel-cut Si(111) monochromator (Δ*E*/*E* = 1.4 × 10^−4^) and can be focused by three independent sets of Be compound refractive lenses (CRLs) located at 36 m, 52.2 m and 56.3 m from the source (Jankowski *et al.*, 2023 ▸). The third focus stage, shown in Fig. 1 ▸, consists of an array of nine Be CRLs of radius 100 µm, and leads to a beam size of 5.4 µm × 1.6 µm (H × V, FWHM) at the sample position 61 m downstream of the source.

**Figure 2:**
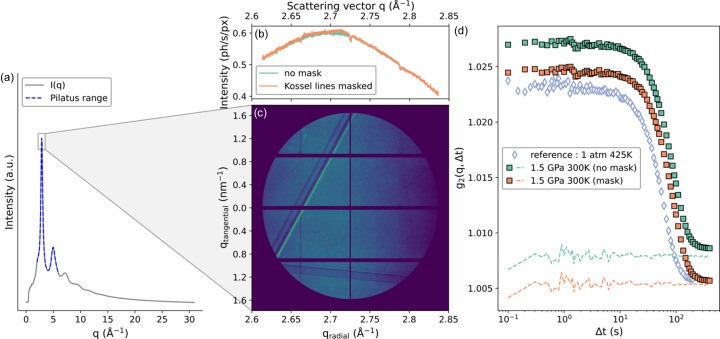
(*a*) Diffracted intensity of a Pt_42.5_Cu_27_Ni_9.5_P_21_ metallic glass, taken at 300 K and 1 atm at the ID15a beamline of ESRF. The range covered by the diffraction detector is shown by the blue dashed line. The range covered by the XPCS detector is indicated by the box on the first peak. (*b*) Scattered intensity after azimuthal integration of the scan-averaged intensity within the XPCS detector, considering all active pixels in the detector (orange solid line), or after masking the two large Kossel lines (green solid line). (*c*) The averaged two-dimensional pattern measured by the XPCS detector during a 700 s scan. (*d*) *g*_2_(*q*,Δ*t*) functions obtained from the same scan, with and without masking the Kossel lines (full symbols), and the *g*_2_(*q*,Δ*t*) function generated by the diamonds and PTM, obtained when aiming the X-ray beam out of the sample (dashed lines). The *g*_2_(*q*,Δ*t*) function of a similar metallic glass obtained in a furnace is shown for comparison (empty symbols).

The large sample-to-detector distance is constrained by the speckle size *d*_speckle_ ≃ λ*R*/*s*, *R* and *s* being the sample-to-detector distance and the X-ray spot size, respectively, which must match the detector pixel size to resolve the speckles and collect the highest scattered intensity for the optimum SNR (23). For these experiments, data are collected by a CdTe EIGER 4M detector from DECTRIS, with a pixel size of 75 µm × 75 µm. The correlation functions are calculated from time series of the scattering patterns [Fig. 1 ▸(*d*)] using the event correlator described by Chushkin *et al.* (2012 ▸). The detector covers a limited range in scattering vector *q* and the correlation functions are calculated and averaged over azimuthal sectors in *q* where the scattering intensity is considered constant. In order to characterize the structure of the amorphous material and the dynamics simultaneously, an additional PILATUS 300k detector is placed downstream from the sample for standard X-ray diffraction. The diffraction pattern, shown in Fig. 1 ▸(*c*), spans a sufficiently wide range in *q* to cover the first two diffraction peaks, which allows us to follow and analyse the evolution of the structure factor during the XPCS measurements, as shown in Fig. 2 ▸(*a*).

To generate HP, a membrane-based DAC is employed [Fig. 1 ▸(*b*)]. Two diamonds facing each other form an experimental volume of dimensions 70 µm × 300 µm (height × diameter), contained radially with a metallic gasket previously deformed at the target pressure. In this DAC the pressure is generated by a metallic membrane. One diamond is attached to a mobile piston, driven by the metallic membrane which inflates when pressurized. The relative change in diameter from the membrane (50 mm wide) to the culet size of the diamond (600 µm wide) generates pressures in the multi-gigapascal range for input pressures within 0 bar to 100 bar. The membrane pressure is controlled remotely by an automatic pressure driver (PACE 5000, Druck) with a precision of 10^−3^ bar. A pressure-transmitting medium (PTM) filling the experimental chamber ensures a hydro­static pressure on the sample, although deviatoric shear stress inevitably appears above the solidification of the PTM [2–20 GPa at 300 K depending on the PTM (Klotz, Chervin *et al.*, 2009 ▸)]. In this study, we used a Le Toullec-type membrane-driven DAC equipped with 3.1 mm-wide, 1.7 mm-high, 600 µm culet-size diamonds, and samples of about 50 µm in size.

The experimental chamber also comprises a fiducial marker for the pressure determination. We used ruby spheres with a controlled Cr amount, as the evolution of the ^2^E → ^4^A_2_ transition wavelength is well calibrated under pressure (Shen *et al.*, 2020 ▸). Pressure determination is also possible from the cell parameter of pure compounds with a known equation of state, provided that enough reflections are visible within the XRD detector across the full pressure range. An optical bench which translates in and out of the beam path provides the 405 nm laser excitation and collection for the ruby signal through a 10× magnification objective, dispersed by a 600 lines mm^−1^ grating and recorded by a Peltier cooled-CCD camera for a final resolution of 0.1 nm. This optical bench also provides a white-light illumination of the DAC volume collected by a camera for an online diagnostic of the sample environment as an alternative to X-rays.

The effect of the DAC, or more specifically the diamonds, on the speckle visibility is shown in Fig. 2 ▸. In this figure, we plot the data obtained for a Pt_42.5_Cu_27_Ni_9.5_P_21_ metallic glass, compressed *in situ* at 1.5 GPa, using a 4:1 methanol:ethanol mixture as the PTM, which ensures hydro­staticity at the applied pressure. The total scattered intensity during the scan (7000 frames, 0.1 s acquisition time) in the detector centred on *q* = 2.73 Å^−1^ is visible in Figs. 2 ▸(*b*) and 2 ▸(*c*). It features the top of the broad first diffraction peak of the glass, plus straight lines which correspond to the Kossel lines from the monocrystalline diamonds (Faigel *et al.*, 2016 ▸; Gog *et al.*, 1995 ▸).

The dynamics of the glass is represented by the *g*_2_(*q*,Δ*t*) function obtained through the correlation of the 7000 frames in Fig. 2 ▸(*d*). One curve is obtained by considering the full active area of the detector [all the active pixels in Fig. 2 ▸(*c*)], the other with the pixels corresponding to the Kossel lines excluded from the correlation calculation. A similar well defined *g*_2_(*q*,Δ*t*) function is obtained with a high SNR in both cases, only shifted vertically. This indicates that the Kossel lines only add a static contribution to the correlation function, and do not affect the probed dynamics of the glass. However, in the case of slow dynamics, full decorrelation is not always resolved in the duration of the measurement. If the reference value for complete decorrelation is affected by static correlations such as the Kossel lines (or other spurious scattering), the corresponding constrained parameter in the modelling of the data can be erroneous. In this case, the static reference signal acquired next to the sample can be used to constrain the modelling of the data with the correct baseline.

The dashed lines correspond to data taken while the X-ray beam points on the diamond only, outside the sample. Contributions to this flat correlation function include X-rays scattered by the PTM and the two diamonds. Since the used PTM is a liquid (alcohol), the timescale of the ISF of the alcohol mixture is many orders of magnitude faster than our temporal resolution of 0.1 s, so only the incoherently scattered X-rays contribute to the dashed flat signal of Fig. 2 ▸(*d*). Scattering from diamonds is purely static, so the dashed lines represent the baselines of the previous *g*_2_(*q*,Δ*t*) functions, and allow for a complete parametrization of the data modelling. To compare the effect of the DAC on this reference correlation value, we plot in the same panel the *g*_2_(*q*,Δ*t*) function obtained in a furnace (*i.e.* no DAC) for a Pt_42.5_Cu_27_Ni_9.5_P_21_ metallic glass sample from the same batch, at a temperature where the timescale of the *g*_2_(*q*,Δ*t*) function is similar. Once the mask is applied, both *g*_2_(*q*,Δ*t*) functions have a comparable baseline value after full decorrelation, which demonstrates that the contribution from the DAC to the correlation function is not significant when crystalline features of the scattering signal are properly masked.

A similar amplitude, also called contrast, of the *g*_2_(*q*,Δ*t*) functions obtained *in situ* at 1.5 GPa and at 1 atm also demonstrates the absence of any degradation of the coherence of the X-ray beam by the diamonds. The slightly higher contrast at 1.5 GPa originates either from geometrical effects as the sample gets thinner with pressure or from pressure-induced structural changes in the material through the non-ergodicity parameter *f*_*q*_. Overall, the diamonds and the PTM do not have a significant impact on the contrast of the correlation functions. The relaxation phenomena behind the atomic dynamics, and therefore behind the long-time decay of the ISF in Fig. 2 ▸(*d*), are described in detail by Cornet *et al.* (2023 ▸). They show that a compression at moderate pressures leads to fast intermittent dynamics, and to physical ageing at higher pressure. This two-step scenario where the nature of the dynamics changes as a function of pressure supports a rejuvenation and strain-hardening observed macroscopically (Pan *et al.*, 2018 ▸, 2020 ▸) and is consistent with numerical simulations showing the existence of a pressure-induced second local minimum after the first coordination shell which disappears at higher pressure (Ngan *et al.*, 2021 ▸).

### 2.3. Third- and fourth-generation synchrotron sources

**Figure 3:**
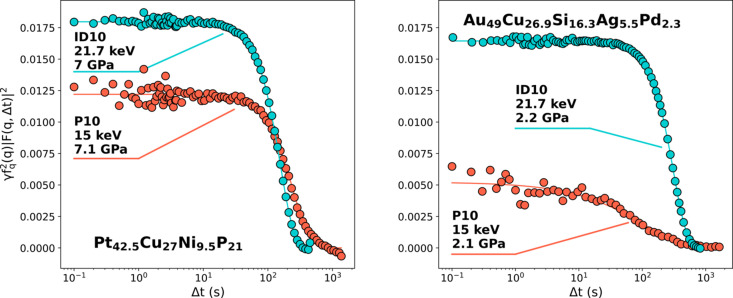
Intensity–intensity correlation function measured on Pt_42.5_Cu_27_Ni_9.5_P_21_ and Au_49_Cu_26.9_Si_16.3_Ag_5.5_Pd_2.3_ ribbons at 7 GPa and 2 GPa, respectively, at the P10 beamline of the PETRA III source, and at the ID10 beamline of the ESRF. For both samples, the ribbons were prepared from the same batch, and thinned to the same thickness (around 15 µm).

Although the new coherent properties of fourth-generation synchrotron sources are ideal for high-pressure–high-temperature XPCS (HPHT-XPCS) studies, it is important to point out that HP-XPCS can still be performed on some third-generation synchrotron sources, such as PETRA III, where XPCS can be performed up to 15 keV. This is highlighted in Fig. 3 ▸, where we compare *g*_2_(*q*,Δ*t*) functions obtained under pressure at room temperature on two different metallic glasses at the P10 beamline at the PETRA III synchrotron source (Hamburg, Germany) with data obtained at the ID10 beamline at the ESRF at similar pressures, for samples with identical thicknesses. The SNR in the *g*_2_(*q*,Δ*t*) function at 15 keV remains satisfactory, especially for the Pt_42.5_Cu_27_Ni_9.5_P_21_ glass, also demonstrating the feasibility of HP-XPCS at third-generation sources. However, a drop in contrast is visible in the results obtained at 15 keV compared with that obtained at 21 keV. The contrast drop is even more pronounced when considering the respective beam sizes: 2.3 µm × 1.4 µm at 15 keV against 5.4 µm × 1.6 µm at 21 keV (H × V, FWHM), which should translate to a higher speckle contrast at P10 for an identical configuration. As the photon energy effect on the overall contrast depends on many factors, such as a change in the longitudinal coherence length, the speckle size or a lower scattering angle for the same *q* value, the comparison of data acquired at different energies is not straightforward. The energy shift also leads to a different relative strength in the scattering of the sample and the diamonds, which also affects the contrast. In addition to this multifactorial variation of the contrast with the energy, one must also consider the differences of the experimental setup. In particular, the P10 beamline is equipped with an Si based detector, while the scattering patterns at ID10 are recorded with a CdTe based detector. The quantum efficiency of the former decreases from 98% at 8 keV to 47% at 17.5 keV, while that of the CdTe based sensor remains above 90% below 26 keV, effectively leading to a higher contrast for CdTe based detectors in high-energy XPCS. Finally, these detectors can discriminate between photons with energies below or above a defined threshold, but this threshold should not exceed 80% of the used photon energy. Given the multiple edges in the atomic form factors of Pt and Au between 13 keV and 14.5 keV, the loss of contrast is also explained by the contribution of the fluorescence photons that cannot be filtered out in Si based detectors when working at 15 keV, but can be excluded in CdTe detectors working at 21.7 keV.

This loss of contrast can become a limiting factor. This is particularly true for HP-XPCS studies in the supercooled liquid state, where the contrast diminishes even further (Amini *et al.*, 2021 ▸).

### 2.4. Pressure and temperature stability

Any movement of the sample position can induce an artificial decorrelation of the successive collected diffracted patterns, as the illuminated volume changes and a new configuration of scatterers is probed. If the timescale of the sample drift is faster than or comparable with that of the atomic motion, the probed dynamics correspond to this sample displacement (Busch *et al.*, 2008 ▸). Stability is therefore critical as it can be difficult to disentangle artificial from intrinsic dynamics as, for instance, in the case of a continuous drift. The horizontal or vertical beam size, *L*_H,V_, in XPCS measurements is typically around a few micrometres, and glassy dynamics have relaxation times (τ) spanning from hundreds of seconds to hours at room temperature and atmospheric pressure (Ruta *et al.*, 2012 ▸; Giordano & Ruta, 2016 ▸), to the sub-second timescale in the supercooled liquid state (Amini *et al.*, 2021 ▸). Therefore, XPCS requires a sample stability better than ∼10% × *L*_H,V_/τ, which can be difficult to obtain in a DAC, where a slow pressure stabilization usually takes place.

**Figure 4:**
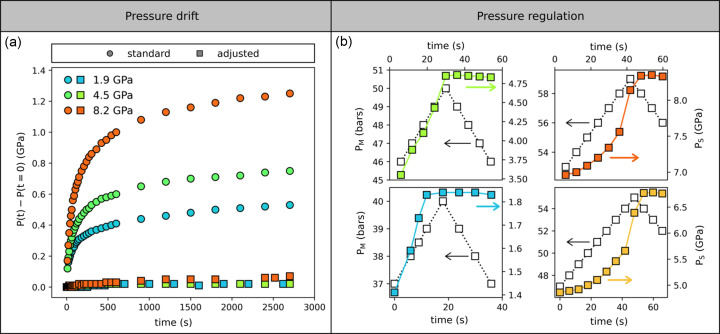
(*a*) Evolution of the pressure measured on a ruby sphere after stabilization of the membrane pressure for different nominal pressures, with (squares) or without (circles) adjusted protocol. (*b*) Evolution of the sample pressure (*P*_S_) after decreasing the membrane pressure (*P*_M_) by 4 bar to 5 bar, at the nominal pressures of 1.8 GPa, 4.8 GPa, 6.7 GPa and 8.3 GPa.

This is shown in Fig. 4 ▸, where we report the pressure stabilization in a DAC after reaching a set point on the membrane pressure. All data were obtained in a DAC equipped with 600 µm diamonds (culet size), stainless steel gaskets, a 4:1 methanol:ethanol mixture as the PTM and a similar rate of 0.1 bar s^−1^ on the membrane. The pressure measured within the cell at the moment the membrane pressure is fixed is given in the caption, and we measure the subsequent evolution of pressure inside the DAC as a function of time. Without regulation [Fig. 4 ▸(*a*), circles], both the time needed for equilibration and the amplitude of the pressure drift increase with the nominal pressure, with a drift larger than 1.3 GPa visible for a nominal pressure of 8.2 GPa. More importantly, equilibrium is still not reached in an hour at this pressure, which precludes any XPCS measurement on this timescale. A possibility to improve the timescale and intensity of the pressure equilibration is to decrease the pressure slightly in the membrane after reaching the set point, as illustrated in the right panel of Fig. 4 ▸. The pressure within the cell is measured while the pressure on the membrane reaches the set point and decreases immediately after (empty symbols). It appears that the sample pressure decreases by a maximum of 0.03 GPa for a reduction of the membrane pressure by 4 bar at all pressures. The long-term evolution of the pressure with this dedicated protocol is shown together with the initial pressure drift in Fig. 4 ▸(*a*) (squares): an obvious reduction of the pressure variation during the equilibration is visible, making XPCS measurement possible almost as soon as the pressure is reached. The evolution of the atomic dynamics under pressure in a Pt_42.5_Cu_27_Ni_9.5_P_21_ metallic glass shows how this can be critical as, at low pressure (<1 GPa), a variation in pressure of only 0.1 GPa leads to an acceleration of the dynamics by a factor of two (Cornet *et al.*, 2023 ▸). Therefore, only a dedicated pressure protocol allows time-resolved XPCS measurement under pressure in the 0–10 GPa range tested here, and to pressures up to 30 GPa with carefully selected PTMs (where the pressure standard deviation σ_P_ of 0.1 GPa in He is still reasonable for high shear modulus materials).

As for standard X-ray diffraction or spectroscopy techniques, it is also possible to change the temperature along with pressure, opening the possibility to perform HPHT-XPCS. Usual means to control the temperature of the samples inside a DAC are laser-heating (Anzellini & Boccato, 2020 ▸) and internal (Heinen *et al.*, 2021 ▸; Mijiti *et al.*, 2020 ▸) and external resistive heating (Santoro *et al.*, 2020 ▸), the latter being the most limited in terms of temperature range and heating rates, but also the most stable in terms of thermal fluctuations and consequently the best choice for HPHT-XPCS.

**Figure 5:**
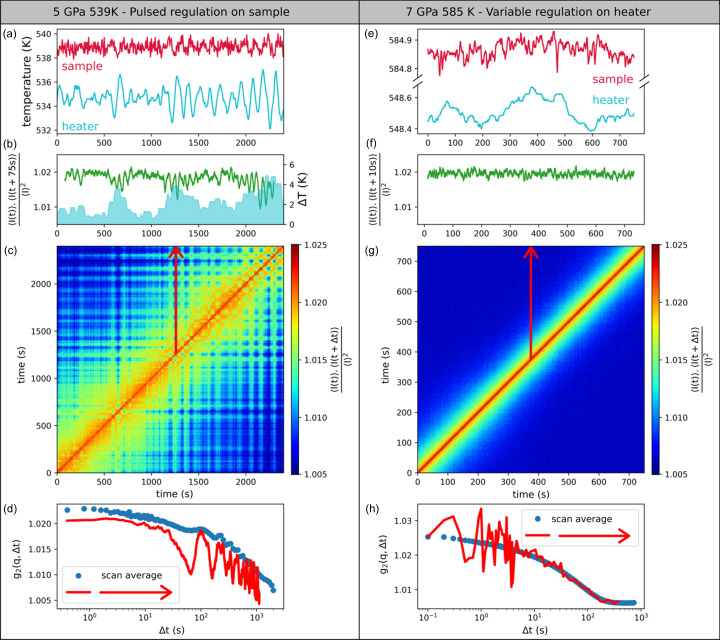
Effect of the temperature regulation on the sample stability. Left: temperature regulation of the sample temperature, pulsed power on the heater. Right: temperature regulation on the heater temperature, variable power on the heater. (*a*, *e*) Furnace and sample temperatures, measured by two distinct thermocouples. (*b*, *f*) Correlation values with fixed delay times Δ*t* of 75 s and 10 s (green), and the amplitude of the variation of the furnace temperature. (*c*, *g*) TTCFs acquired during the temperature monitoring of (*a*, *e*). (*d*, *h*) *g*_2_(*q*,Δ*t*) correlation functions (red line) from a fixed reference frame (see the red arrows on the TTCF) compared eith those (blue dots) averaged over the measurement time interval.

As for pressure, a high thermal stability of the whole system is needed to extract reliable dynamics: the stability of the sample temperature is a necessary but not sufficient criterion for HPHT-XPCS, as an excellent temperature regulation could anyway be accompanied by variation in the sample position at the micrometre scale which would partially (or globally) decorrelate the XPCS signal. The mechanical stability at the micrometre level is thus a requirement of XPCS studies, which is often not necessary for other techniques, like for instance HP-XRD experiments on glasses. To connect the thermal fluctuations to their effects on the probed dynamics, we report in Fig. 5 ▸ the evolution of the temperatures measured on the heater resistance and at the sample position, and the instantaneous intensity–intensity correlation map *C*(*t*_1_, *t*_2_), called the two times correlation function (TTCF) and defined by

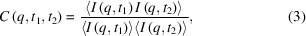

which represents the correlation between the intensity *I* measured at a given wavevector **q** of speckle patterns collected at two distinct times *t*_1_ and *t*_2_, and the average is performed over all pixels of the detector corresponding to the same *q* = |**q**|. By averaging correlation values (*C*_I_) over all identical delay times Δ*t* = *t*_2_ − *t*_1_, one obtains the intensity–intensity correlation function *g*_2_(*q*,Δ*t*) = 〈*C*_*I*_(*q*, *t*,Δ*t*〉_*t*_. Focusing on the TTCF in the right panel of Fig. 5 ▸, the main diagonal corresponds to *t*_1_ = *t*_2_ = *t*, and represents the reference time of the laboratory. The continuous decrease from high (red) to low (blue) correlation values out from this main diagonal corresponds to the *g*_2_(*q*,Δ*t*) function defined in equation (1) for increasing reference times Δ*t* = *t*_2_ − *t*_1_, the width of the red contour is proportional to the characteristic relaxation time τ.

In the left panel of Fig. 5 ▸, we report the temperature stability at 5 GPa and 539 K for a DAC equipped with an external resistive heating collar, where the temperature regulation is performed on the sample temperature (measured by a thermocouple in contact with the back side of the diamond) in a pulsed mode (0% or 100% heating power), *i.e.* with a standard commercial setup usually employed in HP and HT studies.

The sample temperature is stable up to 1 K, while the temperature on the heater fluctuates with a peak-to-peak amplitude reaching 5 K. The TTCF recorded simultaneously on Pt_42.5_Cu_27_Ni_9.5_P_21_ metallic glass is also shown on the same temporal axis, and clearly shows a hashed texture for the correlation values. This effect arises from micrometre movements of the sample following the abrupt thermal expansions of the heater due to the pulsed regulation. This movement of the sample position is strictly verified when we compare the amplitude of the oscillation of the furnace temperature Δ*T* (cyan curve) with the correlation values obtained with a fixed delay time of 75 s, where the changes in the correlation values are maximal (green curve): large oscillations in 〈*I*(*t*)〉〈*I*(*t* + 75*s*)〉/〈*I*〉^2^ and maxima in Δ*T* are simultaneous.

In Fig. 5 ▸(*f*), we report the temperature stability and TTCF obtained with the same resistive heating sleeve setup on a similar Pt_42.5_Cu_27_Ni_9.5_P_21_ glass at 7 GPa and 585 K, with a dedicated power supply and regulation performed on the heater element with an adjustable heating power after an optimization of the proportional integral derivatives (PIDs) parameters. Not only is the temperature stability enhanced to a peak-to-peak amplitude below 0.1 K, but the sample stability is now achieved at the micrometre scale. The corresponding smooth TTCF demonstrates the feasibility of HTHP-XPCS with an intensity stability characterized by a standard deviation of 7 × 10^−4^ [Fig. 5 ▸(*f*)]. Note that the effect of the sample instability is better highlighted in the TTCF as it is smeared out in the averaged *g*_2_(*q*,Δ*t*) as shown in the bottom panels of Fig. 5 ▸, where we compare the *g*_2_(*q*,Δ*t*) averaged over the full time series with the *g*_2_(*q*,Δ*t*) corresponding to the temporal evolution of the intensity–intensity correlation value from a fixed reference frame (see the red arrows on the TTCF). In the first scenario where the temperature regulation is not optimized, the oscillations appear clearly on the single *g*_2_(Δ*t*) function, but smear out on the averaged correlation function. In the case where the temperature regulation is optimized, dynamics are homogeneous over the duration of the scan, and the two *g*_2_(*q*,Δ*t*) functions coincide as in Fig. 5 ▸(*h*). Therefore, the consistency of the results should always be verified from the TTCF with a high-intensity resolution as lower X-ray fluxes can hide specific features in the TTCFs.

Importantly, the pressure on the sample changes with temperature, and a membrane-driven DAC with a remote-controlled pressure inlet combined with continuous monitoring of the sample pressure is necessary to maintain pressure stability on heating or cooling through a manual compensation of the temperature-induced variation of the sample pressure.

Finally, temperature also promotes the creep deformation of the gasket under pressure, which potentially translates to sample movements if the sample is in contact with the gasket, and to a pressure drift within the measurement. The first issue disappears when the sample is positioned in the centre of the experimental volume, with no contact with the gasket. Regarding the pressure drift, the protocol described in Fig. 4 ▸ leads to pressure uncertainties lower than 0.2 GPa, even at temperatures as high as 630 K.

### 2.5. Pressure-transmitting medium

**Figure 6:**
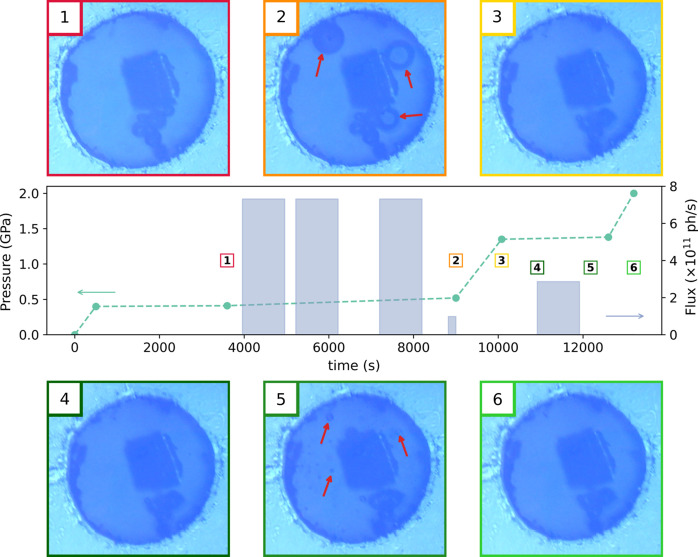
Instantaneous flux on the sample and evolution of the pressure measured on a ruby sphere, with micrographs of the experimental chamber in the gasket. The time of acquisition for each micrograph is represented in the central panel by their corresponding labels. Red arrows highlight the position of bubbles.

The choice of the PTM depends on the pressure–temperature path taken during the measurement, as the stress exerted on the sample must remain hydro­static to avoid plastic flow within the probed material. Depending on the pressure and temperature range investigated, a specific PTM is chosen considering its degree of hydro­staticity (Klotz, Chervin *et al.*, 2009 ▸; Klotz, Paumier *et al.*, 2009 ▸) or its phase diagram (Young *et al.*, 1987 ▸; Datchi *et al.*, 2000 ▸; Vos *et al.*, 1991 ▸). In addition to the strict hydro­staticity constraint, molecular PTMs can also be affected by the strong X-ray beam of the new generation of synchrotron sources. In particular, the 4:1 methanol:ethanol mixture shows degassing under irradiation at low pressure, as shown in Fig. 6 ▸. The micrograph of the loaded cell at *P* = 0.45 GPa shows the position of the sample and of several rubies within the 300 µm hole of the stainless-steel gasket (label 1). After successive scans corresponding to a dose of 5.4 × 10^9^ Gy, we observe the apparition of three bubbles, clearly visible in the new micrograph (label 2), which are resorbed when the pressure is increased to 1.35 GPa (labels 3 and 4). The same pattern appears at this new pressure step for an even lower dose: numerous small bubbles are visible in the micrograph taken after an additional deposited dose of 6.5 × 10^8^ Gy (label 5), which are again resorbed when the pressure is increased to 2 GPa (label 6). No bubbles appeared above 1.4 GPa, even for doses up to one order of magnitude above those considered here.

The primary effect of this degassing is a lack of stability on the sample position. This in turn leads to artificial partial or full decorrelations and unphysical *g*_2_(*q*,Δ*t*) functions. As the intrinsic dynamics of a glass can also be intermittent (Evenson *et al.*, 2015 ▸; Luo *et al.*, 2020 ▸), it is necessary to control the experimental volume before and after each scan to discard artificial sources for irregular dynamics within the glassy samples. Moreover, the apparition of bubbles in the 4:1 methanol:ethanol mixture implies broken bonds in the alcohol molecules due to impinging X-rays. Not only does this lead to bubbles and the loss of sample stability mentioned above, but this also implies the creation of free radicals, which could later react with sample. At low pressure (<2 GPa), it is therefore necessary to control the irradiation levels with an alcohol PTM to mitigate undesirable effects. Finally, the irradiation-induced degassing of the PTM stresses the importance of the online monitoring of the experimental volume, as other issues can appear during the experiment, such as a dendritic crystallization of the 4:1 alcohol mixture at HP and HT, and potential shear stress induced when the sample is pinched by the shrinking gasket.

## 3. Results: stability, time resolution and physical ageing under pressure

Once sample stability has been achieved, it is possible to monitor the internal dynamics of a glass or a supercooled liquid at HP, as shown from our recent results (Cornet *et al.*, 2023 ▸) as well as from the TTCF in Fig. 5 ▸(*g*).

A sample stability compatible with XPCS measurements over long timescales has already been reported at HP and room temperature with a time resolution of 5 s per frame (Zhang *et al.*, 2023 ▸), obtained before the EBS upgrade of the ESRF. The increased intensity of the coherent flux by two orders of magnitude allows measurement with integration time for the individual frames reduced by four orders of magnitude, keeping the SNR constant, reaching the limit of 1 ms imposed by the frame rate of the detector. Thus, the sample stability achieved combined with the high SNR due to the enhanced coherent flux of the ESRF-EBS source opens the possibility for a time-resolved evolution of the dynamics after pressure perturbation and/or temperature perturbation at HP.

**Figure 7:**
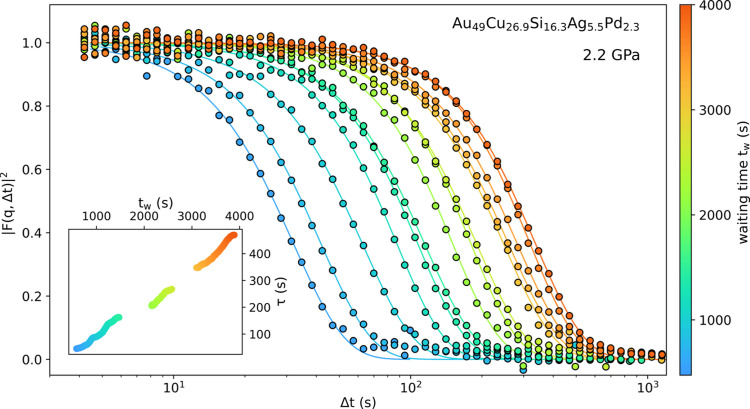
Correlation functions in a Au_49_Cu_26.9_Si_16.3_Ag_5.5_Pd_2.3_ metallic glass at 2.2 GPa as a function of the elapsed time *t*_w_ after setting the pressure. Lines are KWW fits to the data. The inset shows the evolution of the characteristic relaxation times (τ) from the KWW fits as a function of the waiting time *t*_w_.

The interest for time-resolved XPCS under HP is highlighted in Fig. 7 ▸, which features selected |*F*(*q*,Δt)|^2^ functions obtained on an Au_49_Cu_26.9_Si_16.3_Ag_5.5_Pd_2.3_ metallic glass at 2.2 GPa and 300 K, as a function of the waiting time *t*_w_ after the pressure perturbation. These successive ISFs are obtained by binning the TTCF, effectively probing the correlation of stacks of 1000 frames of 0.1 s exposure time with all the subsequent frames of the scan. The SNR of the ISFs remains excellent despite the relatively low number of frames, and a clear trend appears in the figure, where the shift of the ISF to larger times with increasing *t*_w_ indicates a slow-down of the glass dynamics during the isobar. The characteristic relaxation time τ of the ISF can be extracted by fitting the Kohlrausch–Williams–Watts (KWW) function 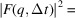 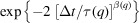 to the data. The final evolution of the characteristic time τ with *t*_w_ is shown in the inset of Fig. 7 ▸ with a time resolution of 100 s (1000 frames × 0.1 s), and a highly detailed curve with a point density of ten data points per second. Therefore, a real time-resolved monitoring of the liquid and glassy dynamics is possible at HP while resolving the ISF from over six orders of magnitude of time, from 10^−3^ s to 10^3^ s.

## 4. Conclusions

Compression in the 0–10 GPa range translates to a density variation from 5–20% in metallic glasses, depending on the bulk modulus of the glass (Zeng *et al.*, 2014 ▸), to 34% in vitreous silica (Wakabayashi *et al.*, 2011 ▸), and to a similar variation in chalcogenide glasses (Mei *et al.*, 2006 ▸). Thus, the possibility to perform *in situ* XPCS under HP and HT truly allows us to investigate the effects of density on the dynamical properties of the structural glasses and their corresponding supercooled liquids, including the glass transition. Some promising applications of HPHT-XPCS include the use of higher-order correlation functions to probe the role of density, and therefore packing, on the dynamical heterogeneities in deeply supercooled liquids (Cipelletti *et al.*, 2003 ▸; Perakis *et al.*, 2017 ▸). The dynamical heterogeneities are variation in time and space of the dynamics in glass-formers, which appear during the enormous increase of viscosity on cooling that eventually leads to the glass transition (Berthier *et al.*, 2011 ▸). XPCS can provide a quantitative estimate of the heterogeneity of these relaxation processes, through the determination of the χ_4_ four-points correlation function (akin to normalized variance) (Perakis *et al.*, 2017 ▸). As such, the development of HPHT-XPCS opens the possibility to monitor the intensity of the dynamical heterogeneities at the glass transition as a function of the liquid density. Another intriguing field that HP-XPCS can cover is polyamorphism, where amorphous systems switch between two distinct states (Tanaka, 2020 ▸). The advance of HP-XPCS at fourth-generation synchrotron sources promises to enhance the foreseen possibilities offered by XPCS regarding polyamorphism. HP-XPCS can also be applied beyond the field of liquids and glasses, as XPCS shows sensibility to the spin and/or charge fluctuations in quantum materials (Shpyrko *et al.*, 2007 ▸). As such, an adaptation of HP-XPCS to low temperatures has potential for HT superconductors applications, where HP promotes the Curie temperature owing to a redistribution of the charges (Jurkutat *et al.*, 2023 ▸).

## Figures and Tables

**Table 1:** Peak energy and brilliance of the first and third harmonic of a single U27 (1.5 m long) undulator at the ESRF ID10 beamlime, before and after the ESRF-EBS upgrade, with the transmission of X-rays through a typical DAC at the corresponding energy

	Old ESRF		ESRF-EBS		
	*E* (keV)	Brilliance [photons s^−1^ mm^−2^ mrad^−2^ (0.1% bandwidth)^−1^]	*E* (keV)	Brilliance [photons s^−1^ mm^−2^ mrad^−2^ (0.1% bandwidth)^−1^]	Transmission
First harmonic	7.93	2.43 × 10^19^	7.22	1.26 × 10^21^	0.5%
Third harmonic	24	8.25 × 10^18^	21.88	6.18 × 10^20^	68%
